# RANTES levels in peripheral blood, CSF and contused brain tissue as a marker for outcome in traumatic brain injury (TBI) patients

**DOI:** 10.1186/s13104-017-2459-2

**Published:** 2017-03-24

**Authors:** Venencia Albert, Arulselvi Subramanian, Deepak Agrawal, Sanjeev Kumar Bhoi, Pooja Pallavi, A. K. Mukhopadhayay

**Affiliations:** 1 Departments of Laboratory Medicine, Jai Prakash Narayan Apex Trauma Center, AIIMS, New Delhi, 110022 India; 2 Department of Neurosurgery, Jai Prakash Narayan Apex Trauma Center, AIIMS, New Delhi, 110022 India; 3 Department of Emergency Medicine, Jai Prakash Narayan Apex Trauma Center, AIIMS, New Delhi, 110022 India

**Keywords:** TBI, Neuroinflammation, RANTES, Chemokine

## Abstract

**Background:**

Traumatic brain injury (TBI) causes activation of several neurochemical and physiological cascades, leading to neurological impairment. We aimed to investigate the level of novel chemokine RANTES in plasma, cerebrospinal fluid (CSF) and contused brain tissue in traumatic brain injury patients and to correlate the expression of this chemokine with the severity of head injury and neurological outcome.

**Methods:**

This longitudinal case control study was performed on 70 TBI patients over a period of 30 months. Glasgow coma scale (GCS) and Glasgow outcome score were used to assess the severity of head injury and clinical outcome. Level of RANTES was quantified in plasma (n = 60), CSF (N = 10) and contused brain tissue (n = 5). Alterations in the plasma levels on 1st and 5th day following TBI were assessed. Patients were categorized as severe (GCS < 8) (SHI), moderate and mild Head injury (GCS > 8–14). 15 healthy volunteers were taken as the control group.

**Results:**

The median plasma RANTES levels were 971.3 (88.40–1931.1); 999.2 (31.2–2054.9); 471.8 (370.9–631.9) for SHI, MHI and healthy control respectively and showed statistically significant variation (p = 0.005). There was no statistical difference in the mean 1st and 5th day RANTES levels for the SHI group. However, admission RANTES levels were significantly higher in patients who died than those who survived (p = 0.04). Also, RANTES levels were significantly higher in plasma as compared to contused brain tissue and CSF (p = 0.0001).

**Conclusion:**

This is the first study of its kind which shows that there is significant correlation of admission RANTES levels and early mortality. Another interesting finding was the significant upregulated in the expression of RANTES in plasma, compared to CSF and contused brain tissue following severe TBI.

## Background

Traumatic brain injury leads to a complex cascade of pathophysiological and neurochemical events. The influx of neuroinflammatory mediators triggered following the primary injury, results in secondary insult to the brain.

Regulated upon activation normal T cells expressed and secreted (RANTES) is a C–C β chemokine (68 a.a.) is a selective chemo attractant of human monocytes and lymphocytes and induces the migration of monocytes, eosinophils, T cells, NK cells, mast cells, and basophils to sites of inflammation and infection [1], are released from multiple sources, predominantly CD8^+^ T cells, platelets, macrophages, eosinophils, fibroblasts, monocytes [2–4].

RANTES stimulates T cells via two discrete pathways, first is a transient Ca^2+^ mobilization by GPCR-mediated pathway leading to cell polarization and migration, second is a sustained Ca^2+^ surge dependent on protein tyrosine kinase (PTK)-mediated pathway resulting in multiple cellular responses including T cell proliferation or apoptosis, release of interleukin 2 (IL-2), IL-5, interferon γ (IFN-γ) and MIP-1β. Other chemokines do not produce these responses. Thus, in addition to inducing chemotaxis, RANTES can act as an antigen-independent activator of T cells in vitro [4, 5].

RANTES and its receptor CCR5 have been linked to numerous pathological conditions in the brain and neurodegenerative diseases [4]. RANTES role in leukocyte infiltration has been established, recently RANTES-mediated systemic inflammatory response, has been associated to chronic infection and augmented microvascular injury in the brain. Suggesting therapeutic utility of targeted modulation of RANTES-dependent pathways [6].

Injury and the resultant inflammation leads to the breakdown of the blood brain barrier (BBB) compromising permeability of circulating immune cells. Production of inflammatory cells including complement activation proteins by astrocytes, neuron and microglia in response to pathological challenge has previously been reported [1]. Subsequent to brain injury, chemokines initiate integrin clustering, recruit lymphocytes to injury sites, and steer them into the brain, ensuing which, these lymphocytes, together with neuronal cells, participate in proinflammatory cytokine-mediated stimulation of endothelial activation and chemokine secretion [7].

RANTES has been reported to play a role in inflammatory brain diseases such as cerebral malaria [8] and scrapie [9]. Owing to the neurotoxic and neuroprotective functions of chemokines, targeted specific therapies for TBI have not yet been developed to affect underlying causes. Previous studies have reported the elevated expression of RANTES in peripheral blood post brain injury in animal models, however whether plasma level of RANTES can predict severity of brain injury in critically injured trauma patients, remains unknown [10]. There have been very few studies in human subjects on the role of RANTES in the pathogenesis of human TBI.

Clinicians desire reliable biomarkers that reflect the immunologic status after acute TBI. Biomarkers that can help navigate personalized therapies, additionally where to measure (blood vs. CSF vs. tissue), as well as when after injury to measure the marker is a challenge [11]. This study intended to investigate the level of novel chemokine RANTES, in plasma, cerebrospinal fluid (CSF) and contused brain tissue in traumatic brain injury patients within 24 h and on day 5 of injury; also to correlate the expression of this chemokine with the severity of head injury and clinical outcome of the patient.

## Methods

### Setting and design

We conducted a prospective longitudinal case–control study (STROBEs criteria followed), in a level 1 trauma care center, for the duration of 30 months (December 2010–May 2013). 70 isolated traumatic brain injury patients (age group 16–65 years), were included in the study and categorized into four groups (n = 15) i.e. (i) severe head injury (SHI) (GCS ≤ 8) who died within 5 days of injury, (ii) SHI who survived beyond 5 days of injury, (iii) moderate and mild head injury (MHI) (GCS > 8–14) who were discharged within 5 days of injury, (iv) MHI who were hospitalized for more than 5 days of injury, following the assessment for injury using tools like Glasgow coma scale, and computed tomography (CT) findings.

**Fig. 1:**
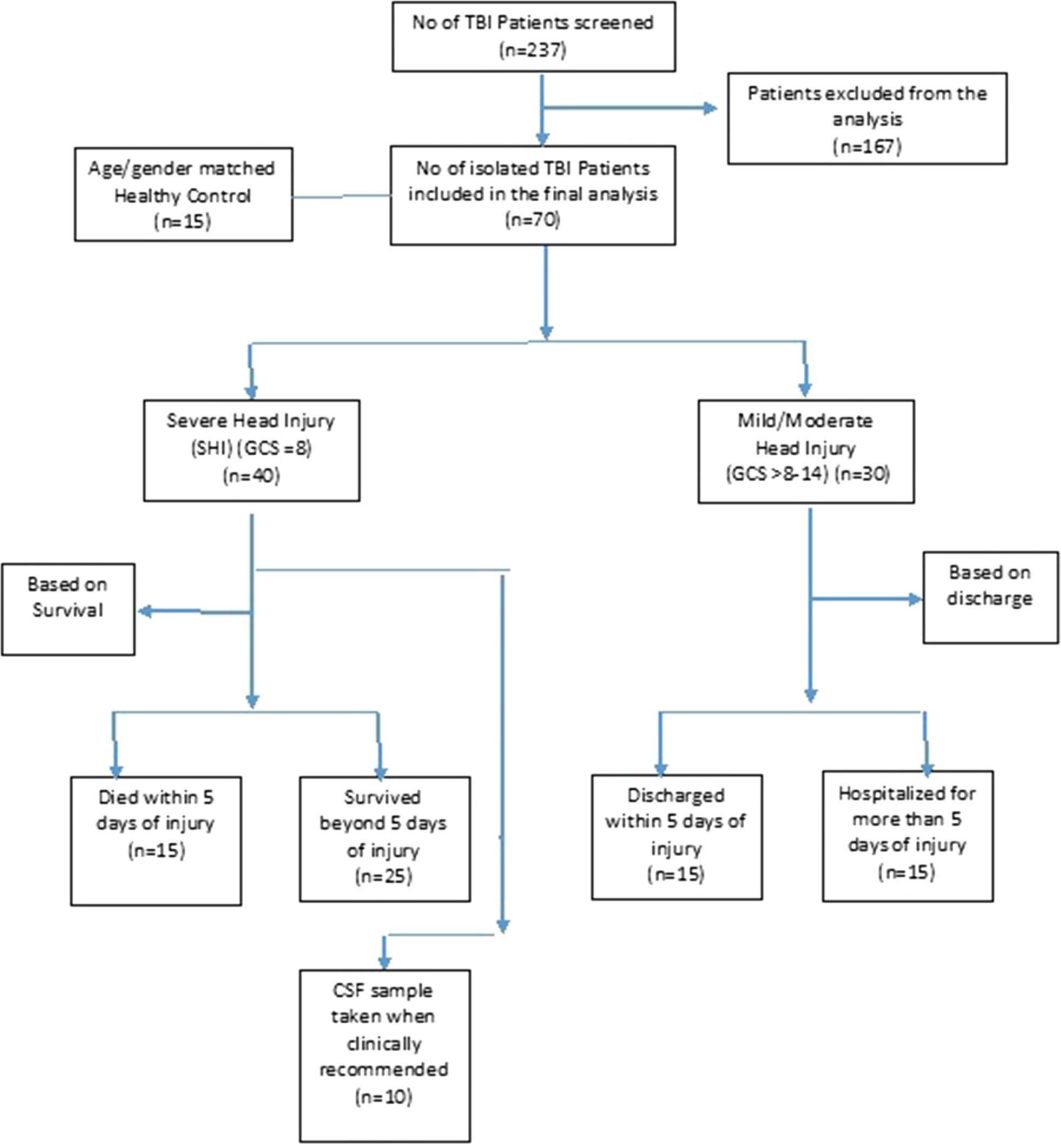
Patient recruitment

Patients with isolated skull fracture, also patients who are immune-compromised or having pre-existing medical problem (diabetes/hypertension/hepatitis) were excluded from the study. Patients admitted after ≥24 h of injury and referred from other institutes were also excluded (Fig. 1).

Age and gender matched 15 healthy controls (HC) were included in the study.

Parameters such as hospital length of stay (HLOS), ICU length of stay (ILOS) and Glasgow outcome score (GOS) at discharge, and development of sepsis (blood culture positivity) and cerebral meningitis (CSF culture positive) throughout hospital stay was recorded.

Peripheral blood was drawn on day 1 and day 5 of injury subsequently for measurement of chemokine RANTES using standard laboratory techniques.

Five contused brain tissue samples were also collected for chemokine analysis, from SHI who survived beyond 5 days of injury, at the time of surgery (within 24 h of injury) from the site of evacuation.

Also, 10 cerebrospinal fluid samples were taken only when clinically indicated from 10 separate patients with SHI the patients, as per the standard scheme of neurosurgical management.

### Diagnosis of traumatic brain injury

TBI was diagnosed based on admission head CT findings [12].

### Sample size calculation

Assuming a common mean ± SD (standard deviation) of 82,840 ± 400 (ρg/ml), one way ANOVA would have 90% power to detect 5% level, a difference in mean RANTES levels, with a sample size in each of three groups (viz. no TBI, mild and moderate, severe TBI) of 10 [12]. Therefore we proposed 15 subjects per group.

Similarly samples for day 5 were studied but only among moderate and severe who had hospital stay for more than 5 days.

### Sample collection and processing

#### Blood

3 ml of intravenous blood samples was collected in EDTA (ethylenediaminetetraacetic acid) vial (1:4) as part of routine blood analysis. Plasma was separated by centrifugation and stored at −20 °C until analysis.

#### CSF

500 μl of cerebrospinal fluid was, collected. Samples were centrifuged at 3000 rpm for 10 min to remove cellular debris and supernatants was decanted and stored at −20 °C until analysis.

#### Contused brain tissue

Tissue were removed and placed in cold (2–4 °C) phosphate buffered saline (PBS) and stored at −80 °C until analysis. Tissue was homogenized as previously described by Hulse et al. [13].

Briefly, the tissue sample was rinsed with cell wash buffer, taken from the Bio-Plex™ Cell Lysis Kit (catalog #171-304012 Bio-Rad; Hercules, CA) once. Tissue was cut into 3 × 3 mm pieces. 500 mM Phenylmethylsulfonyl Fluoride (PMSF) was prepared by adding 0.436 g PMSF (#P-7626 Sigma, St. Louis, MO, USA) to 5 ml dimethyl sulphoxide (#D2650 Sigma, St. Louis, MO, USA) [DMSO], stored in 0.5 ml aliquots at −20 °C. Lysing solution (10 ml) was prepared by mixing the other contents of the Cell Lysis Kit (#171-304012 Bio-Rad) as per manufacturer’s instructions, vortexed gently and set aside on ice, and 40 μl of 500 mM PMSF was added afterwards. To 500 μl of lysing solution, tissue sample was added, tissue disruption was accomplished by drawing the samples up and down through a 1 ml pipette tip (cut back to a 2 mm opening) 20 times, subsequently centrifuged at 4500*g* for 15 min at 4 °C, supernatant was collected.

#### RANTES measurements

RANTES concentrations were measured in duplicates by ELISA (catalog #BMS287/2INST, eBiosciences, Vienna, Austria), following the manufacturer’s instructions.

#### Statistical analysis

Statistical analysis was performed for the comparison of RANTES levels between the groups. Quantitative variables were summarized as mean ± SD or as median (range). Categorical data was expressed as frequency (%) and analyzed using Pearson chi square test. One-way analysis of variance (ANOVA) was applied for comparison between three groups. A p value of ≤0.05 was considered to be statistically significant.

## Results

The study included 30 severe head injury patients (average age 35.1 ± 12.5; 87.9% male; 71.1% road traffic accidents) of which fifteen had subdural hematoma (SDH), five had epidural hematoma (EDH), five had subarachnoid hemorrhage (SAH) and five had multiple contusions. Nineteen mild head injury (GCS 14) and eleven moderate head injury (GCS 9–13) formed the moderate and mild head injury group.

On comparing the baseline parameters for the study groups (severe, moderate and mild head injury and healthy controls) age and gender frequency did not vary. Total leucocyte count was comparatively higher for the SHI group (p < 0.0001).

Nine (30%) SHI patients developed sepsis, of which two died within 5 days of injury, and 4 (13.3%) moderate and mild head injury patients developed sepsis.

Glasgow outcome scale (GOS) at discharge was worse in severe than in moderate and mild head injury group (p < 0.001) (Table 1).

**Table 1:** Baseline parameters

Parameters	Severe head injury (n = 30)	Moderate/mild head injury (n = 30)	Healthy control (n = 15)	*p* value	Bonferroni’s correction
Age	37.5 ± 13.53	36.6 ± 11.49	30.8 ± 6.64	0.17	–
Sex^a^					
Male	25 (83.33)	28 (93.33)	12 (80.0)	0.40	–
Female	5 (16.67)	2 (6.67)	3 (20.0)		
Mode of injury^a^					
RTA	24 (80.0)	20 (66.67)	–	0.56	
Fall	4 (13.33)	7 (23.33)			
Miscellaneous	2 (6.67)	3 (10.0)			
ICU Length of stay^b^	4 (0–25)	1 (0–12)	–	0.0007	
Hospital length of stay^b^	6.5 (1–181)	3 (1–61)	–	0.14	
Systolic BP	123.9 ± 32.10	124.5 ± 18.26	–	0.93	
Time elapsed from injury to sampling (h)^b^	9 (1–24)	6 (1–20)	–	0.11	
Urea (meq/l)	29.5 ± 11.18	27.3 ± 9.77	26.3 ± 5.96	0.52	
Creatinine (meq/l)	0.9 ± 0.3	0.8 ± 0.2	0.7 ± 0.22	0.22	
Sodium (meq/l)	140.7 ± 4.8	140.0 ± 4.04	141.2 ± 2.85	0.63	
Potassium (meq/l)	3.9 ± 0.68	3.8 ± 0.56	4.4 ± 0.42	0.01	SHI versus HC 0.04 MHI versus HC 0.01
Hemoglobin (g/dl)	12.1 ± 3.49	11.6 ± 1.76	13.4 ± 1.69	0.13	
Total leukocyte count (x/cu mm)^b^	15,600 (4400–25,700)	10,500 (850–24,800)	6800 (4300–10,200)	0.0001	
Platelet (/cu mm)^b^	153 (56–338)	150 (67–303)	202 (120–340)	0.10	
Prothrombin time (s)	16.5 ± 4.06	15.2 ± 1.99	15.2 ± 1.84	0.19	
Activated partial thrombin time (s)	29.8 ± 7.51	27.7 ± 4.06	31.5 ± 6.24	0.13	
INR	1.3 ± 0.4	1.2 ± 0.1	1.1 ± 0.01	0.12	
Emergency surgery					
Yes	16	10	–		
No	14	20			
Sepsis					
Yes	9 (30)	4 (13.3)			
No	21 (70)	26 (86.7)			
Outcome^a^					
Alive	14 (46.67)	26 (89.66)	–	0.001	
Dead	16 (53.33)	3 (10.34)			
Glasgow outcome score (GOS)^a^					
Good recovery	0 (0.00)	7 (25)		<0.001	
Moderate disability	5 (17.86)	18 (64.29)			
Severe disability	6 (21.43)	0 (0.00)			
Persistent vegetative	0	0 (0.00)			
Death	16 (57.14)	3 (10.71)			

One-way ANOVA applied; data represented as mean ± SD

*SHI* severe head injury, *MHI* moderate and mild head injury, *HC* healthy control

^a^Frequency (%)

^b^Median (min–max)

 Of the 10 SHI patients (average age 32.7 ± 14.9 years) from whom CSF samples were collected, five had SDH, two EDH, one SAH and two had multiple contusions (Table 2).

**Table 2:** Clinical details of SHI patients (CSF group)

Parameters	CSF samples (n = 10)
Age^b^	32.7 ± 14.9
Sex^a^	
Male	10 (100)
Female	0
Mode of injury^a^	
RTA	6 (60)
Fall	3 (30)
Miscellaneous	1 (10)
ICU Length of stay	10 (1–42)
Hospital length of stay^b^	24.2 ± 9.6
Systolic BP^b^	114.12 ± 36.3
Time elapsed from injury to sampling (h)	8 (5–11)
Red blood cells cells/cumm	110 (23-full field)
White blood cells (0–5 MNC/cumm)	330 (0–800)
Glucose (40–70-mg/dl)	61 (9–98)
Protein (mg/dl)	109.5 (56–865)
Emergency surgery	
Yes	7 (70)
No	3 (30)
Sepsis	
Yes	4 (40)
No	6 (60)
Outcome^a^	
Alive	6 (60)
Dead	4 (40)
Glasgow outcome score (GOS)^a^	
Good recovery	4 (40)
Moderate disability	0
Severe disability	1 (10)
Persistent vegetative	1 (10)
Death	4 (40)

^a^Frequency (%)

^b^Mean ± SD

### Plasma RANTES levels correlation with severity of head injury

**Fig. 2:**
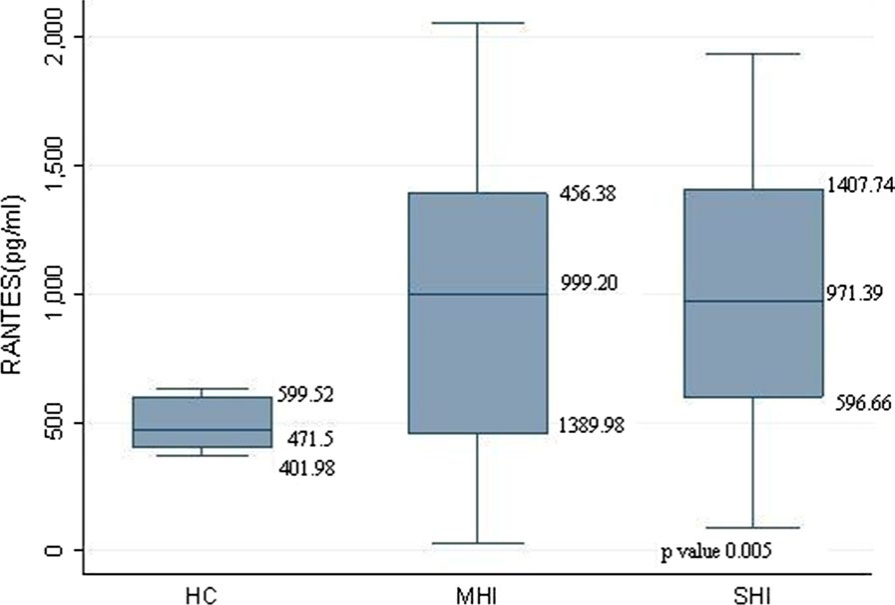
Correlation of plasma levels RANTES in three groups: (i) no head injury, (ii) severe head injury and (iii) moderate and mild head injury. Data represented as median (95–25 percentile). *HC* healthy control, *MHI* moderate and mild head injury, *SHI* severe head injury

RANTES levels within 24 h of head injury was 971.36 (31.23–2054.96) compared to healthy controls 471.8 (370.9–631.9) (p ≤ 0.001). Plasma RANTES levels (ρg/ml) were observed to be 971.39 (88.40–1931.13); 999.20 (31.23–2054.96); for severe, moderate and mild head injury respectively. Overall these variations were observed to be statistically significant (p = 0.005), however further analysis revealed insignificant variation in the plasma RANTES levels between severe and moderate and mild head injury groups (p = 0.85) and significant elevation in the RANTES levels between SHI group and healthy controls, also between moderate and mild head injury group and healthy controls (p ≤ 0.001; 0.01) respectively (Fig. 2).

On admission plasma RANTES levels (ρg/ml) of severe head injury patients who died within 5 days of injury 1155.37 (88.40–1931.13) was comparatively higher than the SHI patients who survived till discharge 742.22 (223.87–1523.66) (p = 0.04).

Only one patient died on the 7th day following injury, on admission RANTES level of 803.99 (ρg/ml) that elevated to 1481.8 (ρg/ml) on day 5.

### RANTES alteration in the plasma at 1st and 5th day following TBI

**Fig. 3:**
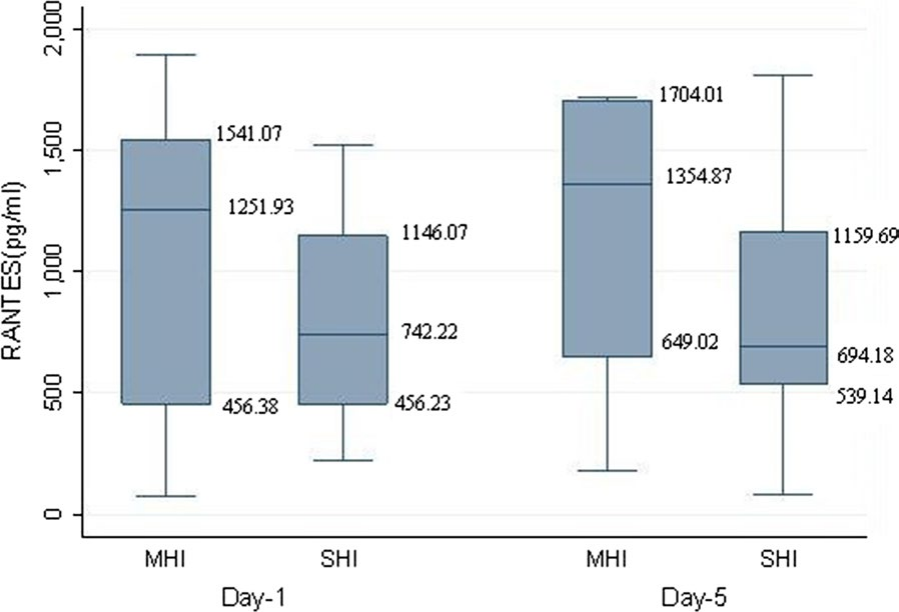
Plasma RANTES (ρg/ml) on day 1 and 5 with the severity of injury in traumatic brain injury patients. Data represented as median (95–25 percentile). *MHI* moderate and mild head injury, *SHI* severe head injury

To evaluate the trend in the plasma levels of RANTES following TBI, we quantified its levels on admission and on the 5th day post injury for 15 patients each in severe and moderate and mild head injury group. The median (min–max) on admission were 742.22 (223.87–1523.6) and 1251 (77.35–1895.58); and on day 5 they were observed to be 694.18 (83.51–1804.73) and 1148.05 (25.63–1718.82) for severe and moderate and mild head injury group respectively (Fig. 3).

Both the 1st and 5th plasma levels were slightly lower in severe group as compared to the moderate and mild head injury group; however this difference was not statistically significant (Table 3).

**Table 3:** Alteration in plasma levels of RANTES at 1st and 5th day following TBI

Groups	RANTES 1st day (ρg/ml)	RANTES 5th day (ρg/ml)	p value
Severe head injury (n = 15)	742.22 (223.87–1523.66)	694.18 (83.51–1804.73)	0.22
Moderate and mild head injury (n = 15)	1251.93 (77.35–1895.58)	1148.05 (25.63–1718.82)	0.35
p value	0.46	0.42	

The difference in the mean day 1 and 5 RANTES levels were calculated for the SHI group, a statistically insignificant decline of 34.7 (ρg/ml) was observed, a similar insignificant decline is reported in the moderate and mild head injury group also (Table 4).

**Table 4:** Mean difference in plasma levels of RANTES on 1st and 5th day following TBI

Groups (n = 15)	Mean difference in RANTES 1st and 5th day plasma levels (ρg/ml)	p value
Severe head injury	34.76	0.91
Moderate and mild head injury	1.03	

### RANTES in cerebrospinal fluid and cerebral contusion in TBI patients

RANTES levels were significantly higher in plasma, lower in contused brain tissue and lowest in CSF. The variation in the chemokine levels were statistically significant across the three groups and within the groups (p = 0.0001) (Table 5).

**Table 5:** Level of novel chemokine RANTES in cerebrospinal fluid of traumatic brain injury patients; and the expression of RANTES associated with cerebral contusion in TBI patients

Type of sample	RANTES (ρg/ml)	p value	Post hoc	p value
Plasma (n = 30)	971.39 (88.40–1931.13)	0.0001	Plasma versus CSF	0.0001
CSF (n = 10)	34.81 (5.43–226.80)		Plasma versus contuse brain	0.003
Contused brain tissue (n = 5)	276.39 (8.47–895.25)		CSF versus contused brain	0.03

Median RANTES levels of 5 SHI patients for both plasma and contused brain were compared, however the variation were not observed to be statistically significant.

Of the ten patients whose CSF was analyzed for chemokine RANTES, four developed sepsis [RANTES levels 376.725 (26.3–226.8) (ρg/ml)] including two who developed cerebral meningitis [RANTES levels 59.76 (26.3–93.1) (ρg/ml)] and six did not develop sepsis throughout their hospital stay [RANTES levels 472.2 (5.4–61.7) (ρg/ml)].

## Discussion

Secondary brain injury is a resultant of a complex cascade of events such as edema, ischemia, excitotoxicity, and inflammation that succeed the initial injury and last throughout acute hospitalization. There is a paucity of clinical studies that corroborate inflammation’s contribution to secondary TBI, which has been well established in experimental conditions.

The following are the major findings of our study on the levels of regulated upon activation normal T cells expressed and secreted (RANTES) a member of the β-chemokine subfamily following traumatic brain injury.

On admission plasma RANTES level was almost twice as high in traumatic brain injury patients in comparison to the healthy control irrespective of the severity of head injury. SHI patients who died within 5 days of injury had higher RANTES levels compared to those who survived. Decline in the plasma RANTES levels by day 5 was observed in severe head injury patients who survived. In SHI patients, plasma levels of RANTES were three times higher than contused brain tissue, within 24 h of injury, however CSF levels of RANTES were significantly lower than contused tissue and plasma. We observed altered RANTES levels in every readily measured compartment, including plasma, CSF, and also in brain tissue posttraumatic brain injury. Our results demonstrate contribution of neuroinflammation in exacerbation of neurologic injury and augmented morbidity and mortality rather than facilitating repair.

Similar to our study, Lumpkins et al. [12]. reported a significantly higher day 1 RANTES level in severe TBI compared with the non-TBI group (mean 1339 vs. 708 ρg/ml, p = 0.046). However, they reported no difference between non-TBI patients and mild TBI patients (708 vs. 751 ρg/ml, p = 0.798). We observed a significant elevation in RANTES levels in the MHI versus healthy group (p = 0.01). Severe TBI group was also found to have a higher RANTES level as compared with the mild/moderate TBI group, but not statistically significant (1139 vs. 751 ρg/ml, p = 0.069) in their study. Similar insignificance for the RANTES levels between SHI and MHI was observed in our study; discordantly we report higher RANTES levels in MHI group as compared with the severe TBI. One possible explanation of this lower cytokine activation could be due to the dilution of the chemokine in the plasma pool via fluid and blood product transfusion.

The decline in the 5th day RANTES levels from 923.1 (409.3–1291.0) on admission to 786.2 (83.5–1804.7) in severe head injury group was observed, likely due to the onset of sepsis, as the association of the down-regulation of RANTES levels with infection is well established in pediatric and adult population [14–16].

Low plasma RANTES level has been established as an independent predictor of mortality in myocardial infarction [17], cerebral malaria [18], inversely we observed a statistically significant association of on admission elevation of RANTES levels following severe traumatic brain to mortality.

Lee et al. [9] stated that central nervous system cells are primarily responsible for the increased chemokine gene expression; reactive were the reported sources of RANTES in scrapie which are triggered to release chemokines and cytokines.

In addition to chemokines, studies also report the elevation of mRNA and protein levels of chemokine receptors following injury. C–C chemokine receptor type 5 (CCR5) mRNA levels were observed to be upregulated in response to the elevated levels of chemokines following injury. CCR5 plays a role in microglial migration towards the lesion site after focal brain injury [19]. Trauma induced activation of astrocytes and microglial cells, may be the predominant cause of upregulation of chemokine levels in the brain following injury. [20].

The patterns of markers of inflammation observed in the peripheral blood tend to be echoed in CSF. Using cerebral micro dialysis, Helmy et al. [21] demonstrated acutely elevated CSF levels of RANTES after severe TBI. They report a stereotyped temporal peak, at least twice the median value of RANTES over the monitoring period on day 1 of cortical injury. CSF RANTES levels in human immunodeficiency virus infected subjects with cognitive impairment was reported to be 95.4 (<5–1442) (ρg/ml) [22].

Sustained elevation of CCL2 [Chemokine (C–C Motif) Ligand 2] of the same β-chemokine family as RANTES was detected in CSF of severe head injury for 10 days after trauma, and in cortical homogenates of mice, peaking at 4–12 h after closed head injury, confirming the significant role of CCL2 in mediating post-traumatic secondary brain damage [23]. We observed a CSF RANTES level of 34.81 (5.43–226.80).

Erikson et al. [24] used multianalyte technology to simultaneously determine the responses of 13 cytokines and chemokine in brain and blood to injections of lipopolysaccharide and path analysis to determine the major relations among these analytes. They report a peak in RANTES levels in brain and serum and concluded that the immune response in the brain is latent compared to that in the periphery, and that expression of these cytokines in the brain likely requires initiation of signaling pathways and transcriptional events within the central nervous system, as previously observed by Tonelli and Postolache [25].

Terao et al. [26] demonstrated a significant elevation in the brain tissue, not plasma, levels of RANTES wild-type mice (WT) subjected to focal cerebral ischemia–reperfusion (I/R), we report an elevation of plasma 837.36 ρg/ml and brain tissue 237.39 ρg/ml RANTES level following injury. Significant elevation in brain tissue levels of RANTES in mice post focal cerebral I/R. BBB dysfunction induced by cerebral I/R was greatly attenuated in RANTES^−/−^ mice suggesting that RANTES directly or indirectly increases BBB permeability.

The results of their study suggested that RANTES plays a major role in the recruitment of both leukocytes and platelets into the cerebral microvasculature after brain I/R. They also reported that circulating blood cells are the likely source of RANTES that mediate the I/R-induced cerebral responses. They observed a persistent elevation of brain tissue RANTES in WT mice transplanted with RANTES(−/−) bone marrow (RANTES > WT) which suggests that non-blood cells (endothelial cells, vascular smooth muscle cells and/or glial cells) are likely to account for the majority of the I/R-induced elevation of brain tissue RANTES, and concluded that of the total RANTES detected in brain tissue of WT-I/R, approximately 40% is derived from blood cells while 60% is derived from non-blood cells.

Hu et al. [27] reported that microglia obtained from fetal and adult brain specimens produced comparable amounts of RANTES suggesting that the capacity to produce this chemokine is acquired early in brain development. Astrocytes, which comprise the major glial cell type within the CNS, were found to be less capable of producing RANTES and anti-inflammatory cytokines regulate the production of RANTES.

## Limitations

The major limitation of this study was that the cerebrospinal fluid samples were not taken from the same patients from whom plasma and contused brain tissue was taken. CSF of ten separate severe TBI patients was analyzed separately. Secondly the Glasgow outcome score of the TBI patients was assessed at discharge and not 1 year following severe TBI.

## Conclusion

This is the first study of its kind which shows that there is significant correlation of RANTES levels within 24 h of injury and early mortality in isolated severe TBI patients. Plasma RANTES was significantly higher in TBI patients irrespective of the severity of injury, in comparison to healthy control. RANTES levels were significantly upregulated in plasma compared to brain tissue, suggesting an inflammatory response to TBI on a local and on a systemic (plasma) level. Our above reported data emphasize the role of neuroinflammation in the escalation of secondary insult which ultimately results in mortality. The pathophysiology of these results should stimulate future clinical trials targeted at alteration of RANTES levels, mitigating secondary brain injury to limit TBI outcomes.

## Abbreviations

RANTES: regulated upon activation, normal T cell expressed, and secreted
TBI: traumatic brain injury
CSF: cerebrospinal fluid
GCS: Glasgow coma scale
GOS: Glasgow outcome score
SHI: severe head injury
MHI: moderate and mild head injury
BBB: blood brain barrier
HC: healthy controls
HLOS: hospital length of stay
ILOS: ICU length of stay
CT: computed tomography
EDTA: ethylenediaminetetraacetic acid
PBS: phosphate buffered saline
PMSF: phenylmethylsulfonyl Fluoride
DMSO: dimethyl sulphoxide
SDH: subdural hematoma
EDH: epidural hematoma
SAH: subarachnoid hemorrhage
CCR5: C–C chemokine receptor type 5
CCL2: chemokine (C–C Motif) Ligand 2
